# Clinical outcomes of nice knot combined with cannulated screws for Beavis type II posterior calcaneal tuberosity avulsion fractures in osteoporotic elderly patients

**DOI:** 10.1186/s13018-025-06385-9

**Published:** 2025-11-03

**Authors:** Peng Lu, Songchuan Su, Xin Li, Liqi Ng, Yusong Liu, Yu Zhou

**Affiliations:** 1 Department of Foot and Ankle Surgery, Orthopedic Hospital, Chonqqing University of Chinese Medicine, Chongqing Orthopedic Hospital of Traditional Chinese Medicine, Chongqing, 400012 China, People’s Republic; 2 Department of Pharmacy, Orthopedic Hospital, Chonqqing University of Chinese Medicine, Chongqing Orthopedic Hospital of Traditional Chinese Medicine, Chongqing, 400012 China, People’s Republic; 3 Institute of Orthopaedic and Musculoskeletal Science, University College London, Royal National Orthopaedic Hospital, Stanmore, London, HA7 4LP England, UK; 4 Postdoctoral Research Workstation, Orthopedic Hospital, Chonqqing University of Chinese Medicine, Chongqing, 400012 China, People’s Republic; 5 Chongqing Institute of Traditional Chinese Medicine Orthopedics, Chongqing, 400012 China, People’s Republic

**Keywords:** Nice knot, Cannulated screws, Beavis Type II posterior calcaneal tuberosity avulsion fracture, Clinical outcomes

## Abstract

**Objective:**

To investigate the surgical technique and clinical efficacy of the Nice knot combined with cannulated screws for Beavis Type II posterior calcaneal tuberosity avulsion fractures in osteoporotic elderly patients.

**Methods:**

A retrospective analysis was conducted on 18 osteoporotic elderly patients with Beavis Type II posterior calcaneal tuberosity avulsion fractures treated at our institution between June 2019 and December 2024. All patients underwent open reduction and internal fixation using a Nice knot combined with cannulated screws. Postoperative assessment included radiographic review supplemented by evaluation of complication rates, fracture healing time, time to full weight-bearing, and the American Orthopaedic Foot & Ankle Society (AOFAS) ankle-hindfoot score.

**Results:**

All 18 patients achieved radiographic union with a follow-up of 12–18 months (mean 13.00 ± 1.85 months). Complications comprised delayed wound healing in two cases, which resolved with regular dressing changes, and periwound paraesthesia in one case that resolved after three months of mecobalamin treatment. The mean time to clinical fracture union was 12.22 ± 1.35 weeks, and the mean time to full weight-bearing was 10.00 ± 1.68 weeks. At final follow-up, the mean AOFAS ankle-hindfoot score was 85.94 ± 6.40, with an excellent/good rate of 77.78%. There were no instances of loss of reduction, implant loosening, or hardware failure.

**Conclusion:**

This preliminary study suggests that the Nice knot combined with cannulated screw technique offers stable fixation, promotes early rehabilitation, and results in a low complication rate in osteoporotic elderly patients with Beavis type II avulsion fractures. However, the retrospective design, small sample size, and absence of a control group limit how broadly these findings can be applied. These preliminary results need validation through future prospective, multicenter randomized controlled trials with larger sample sizes.

## Introduction

Avulsion fractures of the posterior calcaneal tuberosity represent a classic extra-articular fracture pattern, accounting for approximately 1.3% to 2.7% of all calcaneal fractures [1]. Epidemiological studies indicate a higher prevalence among elderly women with osteoporosis or diabetes, with peak incidence occurring around 70 years of age [2, 3]. This injury typically results from either direct trauma to the posterior heel or violent triceps surae contraction during extreme ankle dorsiflexion. In elderly patients with pre-existing osteoporosis, diminished mechanical properties of the calcaneal render it susceptible to failure under forceful Achilles tendon traction, resulting in an avulsion fracture. Due to persistent tendon pull, most posterior tuberosity avulsions demonstrate significant displacement, frequently causing local soft-tissue compromise, including elevated skin tension and irritation, which may progress to flap necrosis. Consequently, surgical intervention is usually required [4, 5]. Nevertheless, no consensus exists regarding the optimal fixation strategy for displaced variants. Crucially, implant selection significantly influences prognosis; inappropriate techniques may precipitate skin necrosis or fixation failure [6, 7].

The management of posterior calcaneal tuberosity fractures remains challenging due to variations in fracture morphology, related injuries, and surgeon preferences. The primary challenge is achieving stable fixation that can resist the strong tensile forces of the Achilles tendon. While isolated cannulated screw fixation offers significant stability with minimal soft tissue dissection, it carries a notable risk of failure in osteoporotic bone due to screw cut-out and superior fragment migration [4, 5]. This concern led us to reinforce the screw fixation with a Nice knot configuration—a technique that provides several biomechanical benefits because of its double-stranded, self-locking, and sliding design, which allows for progressive tensioning and maintains stability under load. The Nice knot has been shown to be biomechanically superior to other sliding knots, such as the surgeon’s knot and the Tennessee slider [8, 9], with its ability to sustain continuous compression being vital for promoting fracture stability and creating an optimal environment for callus formation without compromising vascularity. The clinical potential of this technique is further supported by its successful application in other fracture types, such as severely comminuted patellar fractures [10], Robinson type IIB clavicular fractures [11], and distal clavicle fractures with coracoclavicular ligament disruption [12]. Based on these reported biomechanical and clinical advantages, we hypothesized that the Nice knot could effectively enhance cannulated screw fixation for posterior calcaneal tuberosity avulsions. This study was therefore designed to assess the operative efficacy and clinical outcomes of this combined approach, aiming to provide a potential new treatment option for this challenging injury.

To establish a more effective treatment protocol, this retrospective study analyses clinical data from 18 osteoporotic elderly patients with displaced Beavis Type II posterior calcaneal tuberosity avulsion fractures managed with Nice knot [13, 14] and cannulated screw fixation between June 2019 and December 2024. We present the outcomes below.

## Clinical patient data

This retrospective study was conducted after obtaining approval from the Medical Ethics Committee of Chongqing Orthopaedic Hospital of Traditional Chinese Medicine. Written informed consent was obtained from all participants. The study enrolled 18 elderly patients with osteoporotic Beavis type II avulsion fractures of the calcaneal tuberosity who were treated in the Department of Foot and Ankle Surgery at our institution between June 2019 and December 2024. The cohort consisted of 7 men and 11 women, ranging in age from 62 to 81 years, with a mean age of 69.17 ± 4.88 years. Fracture distribution showed left foot predominance (n = 10) versus right foot (n = 8). Injury mechanisms included low-energy falls (n = 16) and road traffic accidents (n = 2), with all cases being closed injuries. The time from injury to surgery ranged from 1 to 3 days (mean 1.61 ± 0.78 days). Critically, all patients underwent spinal bone mineral density assessment, confirming osteoporosis (T-score > − 2.5 SD). Detailed baseline characteristics are presented in Table 1.

**Table 1:** Demographic Characteristics and Clinical Outcomes of Patients with Osteoporotic Beavis Type II Posterior Calcaneal Tuberosity Avulsion Fractures

ID	Age/gender	Limb (Left/Right)	Injury Mechanism	Comorbidities	Injury-to-Surgery Time (Day)	Time to Full Weight-Bearing (Week)	Time to Fracture Union (Week)	Follow-up Period (Month)	Postoperative Complications	AOFAS
1	A 67-year-old man	Left	Fall	Diabetes, osteoporosis	2	8	10	12	–	92
2	A 68-year-old female	Left	Fall	–	1	8	10	12	–	92
3	A 73-year-old woman	Right	Fall	–	1	12	14	14	–	87
4	A 75-year-old male	Left	Fall	–	1	12	12	12	–	75
5	An 81-year-old man	Right	Fall	–	3	12	14	12	–	78
6	A 71-year-old female	Right	Traffic Accident	–	2	10	12	12	Periwound paraesthesia	75
7	A 68-year-old female	Left	Fall	Diabetes, osteoporosis	2	10	12	12	Delayed wound healing	92
8	A 70-year-old female	Left	Fall	–	1	10	12	12	–	84
9	A 74-year-old female	Left	Fall	Diabetes, osteoporosis	3	12	14	12	–	75
10	A 62-year-old female	Right	Traffic Accident	–	1	8	10	16	–	92
11	A 67-year-old female	Right	Fall	–	1	10	12	12	Delayed wound healing	92
12	A 65-year-old female	Left	Fall	–	1	8	12	18	–	87
13	A 72-year-old female	Left	Fall	Diabetes, osteoporosis	2	12	14	12	–	84
14	A 68-year-old female	Right	Fall	–	1	10	12	14	–	92
15	A 64-year-old male	Left	Fall	–	1	10	12	16	–	87
16	A 73-year-old male	Left	Fall	Diabetes, osteoporosis	3	12	14	12	–	84
17	A 65-year-old female	Right	Fall	Diabetes, osteoporosis	2	8	12	12	–	92
18	A 64-year-old female	Right	Fall	–	1	8	12	12	–	87
Mean ± SD	69.17 ± 4.88				1.61 ± 0.78	10.00 ± 1.68	12.22 ± 1.35	13.00 ± 1.85		85.94 ± 6.40

## Methods

### Ethical approval

This retrospective study was conducted in accordance with the Declaration of Helsinki and was approved by the Medical Ethics Committee of Chongqing Orthopedic Hospital of Traditional Chinese Medicine (Chongqing Orthopedic Hospital of Traditional Chinese Medicines) (GKYYIRB20250301). The requirement for informed consent was waived due to the retrospective nature of the study.

### Surgical method

All procedures were performed under epidural anaesthesia. Patients were positioned prone with soft padding beneath the anterior tibia and ankle to maintain natural knee flexion, ensuring triceps surae relaxation. A pneumatic tourniquet was applied at the proximal thigh after sterile preparation with povidone-iodine solution and draping. A posterolateral longitudinal incision was made commencing 3 cm proximal to the lateral malleolus apex, extending distally across the fracture line. The incision bisected the posterior third between the posterior fibular border and Achilles tendon. Full-thickness dissection proceeded to the periosteum, exposing the fracture site. Debris and hematoma were excised, followed by copious saline irrigation.

**Fig. 1:**
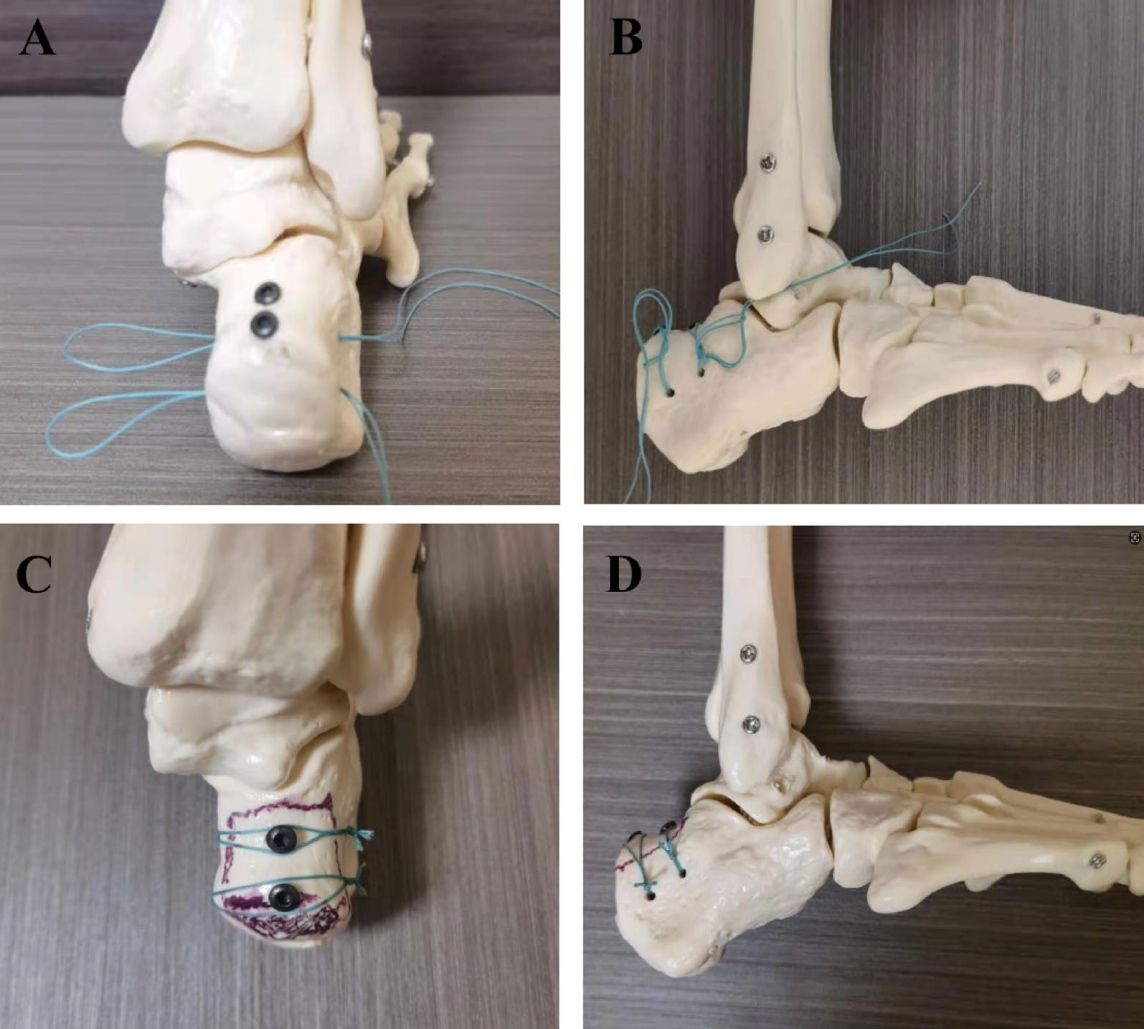
Illustrations and intraoperative photographs demonstrating the key surgical steps. **A**: Screw and Nice knot configuration; **B**: Nice knot tightening; **C**: Lateral view; **D**: Posterior view

Reduction was achieved using Weber reduction forceps under direct vision. Provisional fixation involved inserting 1–2 Kirschner-wires perpendicular to the fracture plane anterior to the Achilles insertion, penetrating the plantar cortex to exit the heel pad. Fluoroscopy confirmed reduction and wire placement. Cannulated drilling was performed along the guidewires with depth control to prevent plantar cortical breach. After depth measurement, 1–2 cannulated screws (4.0 mm diameter) were inserted engaging the plantar cortex, whereupon Kirschner-wires were removed. Subsequently, two 2.0 mm Kirschner-wire tunnels were drilled perpendicular to the calcaneal body, 1–2 cm distal to the fracture line at 1 cm intervals. A medial counter-incision allowed creation of a subcutaneous tunnel connecting both incisions using artery forceps. A doubled Ethibond Excel™ 2–0 suture (Johnson & Johnson) was passed through both osseous tunnels via spinal needle guidance, threaded subcutaneously around the screw heads, and secured using the Nice knot configuration. Ankle range-of-motion testing confirmed construct stability. Closure employed absorbable sutures in layers. Sterile dressings were applied with compression bandaging, followed by anterior slab immobilisation, maintaining the ankle in 20° of plantar flexion. Figure 1 illustrates key technical steps.

### Postoperative management and rehabilitation

A standardized rehabilitation protocol, divided into distinct phases each with specific objectives and interventions, was implemented for all patients following surgery. The protocol was as follows[15–20]:

1. Phase 1 (Postoperative weeks 1–2): The wound dressings were kept clean and dry, with changes performed every 2–3 days. Sutures were removed at two weeks postoperatively. The primary goals during this phase were oedema control and thromboprophylaxis. Specific measures included: elevation of the affected limb; application of cryotherapy to reduce swelling; passive toe flexion and extension performed in multiple daily sets of 3–5 repetitions; and isometric contractions of the quadriceps and gluteal muscles. An X-ray review was conducted at two weeks post-surgery to assess the position of the internal fixation and fracture alignment.
2. Phase 2 (Weeks 2–6): The objectives of this phase focused on preventing soft-tissue adhesions, capsular contracture, and muscular atrophy. Recommended exercises included: active ankle dorsiflexion held at the end range for 20 s; active plantarflexion held at the end range for 20 s; and active inversion and eversion of the ankle performed within pain tolerance in multiple daily sets. Between weeks 2–4, touch-weight bearing ambulation was initiated using elbow crutches with an orthopaedic walker boot, avoiding full weight-bearing. From weeks 4–6, partial weight-bearing of 25–50% body weight was permitted in the walker boot. A follow-up X-ray was obtained at six weeks; CT scanning was performed if necessary to evaluate fracture healing progression. Weight-bearing was gradually increased from 25 to 50% of body weight.
3. Phase 3 (Weeks 6–12): This phase aimed to restore ankle range of motion, improve strength, and promote bone healing. Interventions included: joint mobilisation techniques to enhance mobility of the subtalar, calcaneocuboid, and talonavicular joints; and resistance training using manual pressure or elastic bands. Between weeks 6–8, weight-bearing increased to 50–75% of body weight. Follow-up X-ray or CT imaging at 8–12 weeks guided the transition to full weight-bearing (100%) if satisfactory radiological healing was observed.
4. Phase 4 (Beyond 12 Weeks Postoperatively): The final phase focused on proprioception recovery and gait restoration. Recommended activities included: single-leg stance training on level surfaces, progressing from eyes-open to eyes-closed conditions; centre-of-gravity displacement exercises; and gait re-education aimed at normalising walking. Patients were encouraged to gradually resume normal daily activities and suitable sports participation. This protocol was applied to all patients in the cohort. The transition between phases was based on the elapsed postoperative time and radiographic evidence of early healing, with adjustments made only for individual tolerance to pain and swelling.

### Statistical analysis

All statistical analyses were conducted using R version 4.3.1 (R Foundation for Statistical Computing, Vienna, Austria). Continuous variables were assessed for normality with the Shapiro–Wilk test. Data that follow a normal distribution are expressed as mean ± standard deviation, whereas non-normally distributed data are shown as median (interquartile range). Categorical variables are documented as frequencies and percentages (n, %). Due to the observational, single-cohort, and preliminary nature of this study, the analysis was deliberately limited to descriptive statistics to characterise the patient population and report clinical outcomes, without undertaking comparative hypothesis tests.

## Results

All 18 patients completed follow-up, with a mean duration of 13.00 ± 1.85 months (range: 12–18 months). Clinical fracture union was achieved at a mean of 12.22 ± 1.35 weeks (range: 10–14 weeks). The mean time to full weight-bearing was 10.00 ± 1.68 weeks (range: 8–12 weeks). Postoperative complications occurred in three patients (16.67%), comprising two cases of delayed wound healing that resolved with regular dressings and one case of periwound paraesthesia that improved following a three-month course of mecobalamin. No instances of implant loosening, hardware failure, or loss of reduction were observed.

**Fig. 2:**
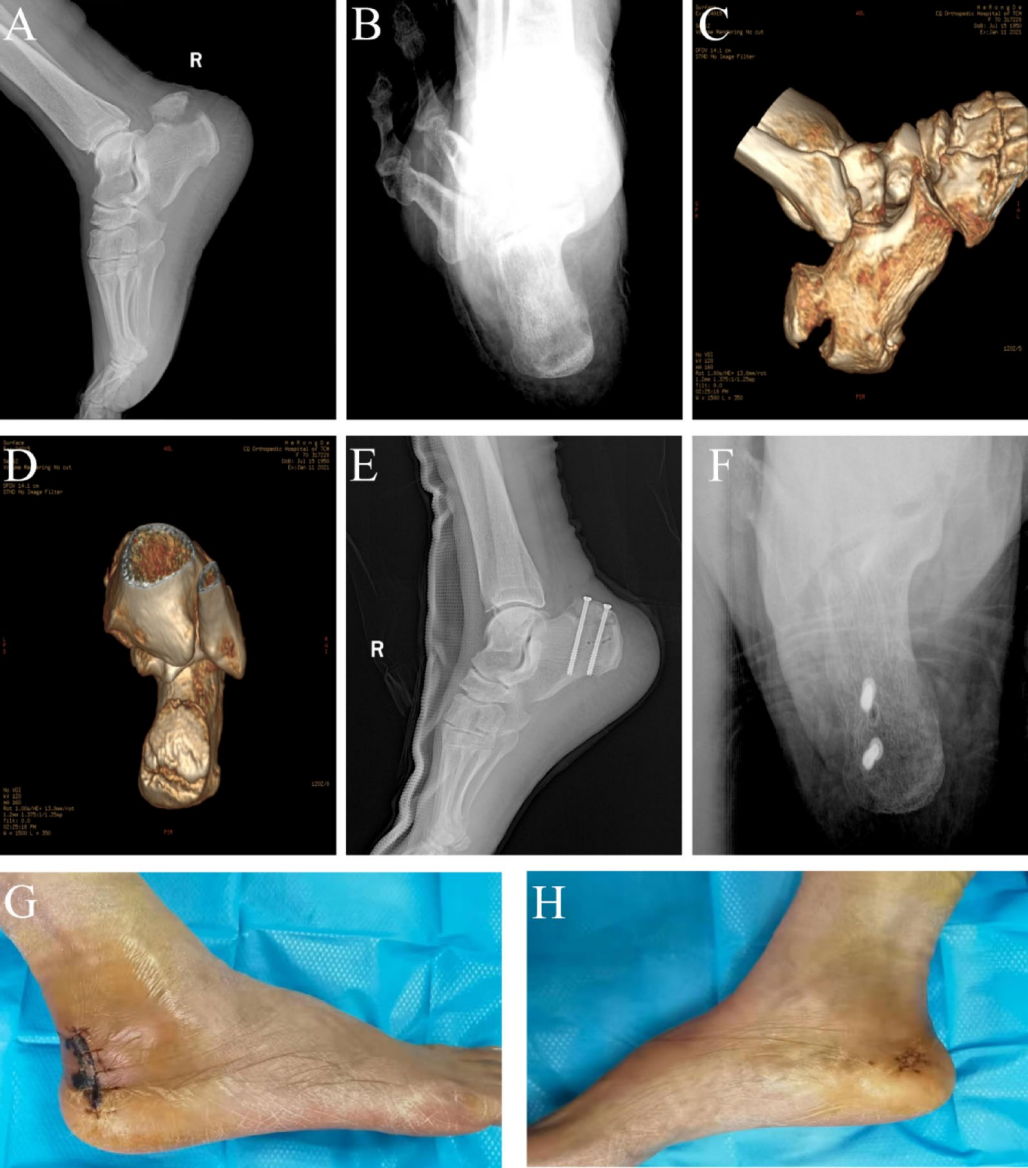
Preoperative imaging and postoperative outcomes in a representative case of Beavis Type II posterior calcaneal tuberosity avulsion fracture. **A**–**B**: Preoperative dorsoplantar and lateral radiographs; **C**–**D**: Preoperative axial and sagittal CT reconstructions; **E**–**F**: 3-month postoperative radiographs demonstrating fracture union; **G**–**H**: Wound appearance at 2-week suture removal

At the final follow-up, functional outcomes were assessed using the American Orthopaedic Foot & Ankle Society (AOFAS) ankle-hindfoot score[21]. This composite outcome measure was chosen because it effectively reflects the morbidity associated with tibiotalar joint stiffness—a common complication following immobilisation in plantarflexion for this injury—and facilitates direct comparison with existing literature on calcaneal fractures. The mean AOFAS score was 85.94 ± 6.40. According to the AOFAS rating system (Excellent: 90–100; Good: 80–89; Fair: 70–79; Poor: ≤ 69), outcomes were graded as excellent in 7 cases, good in 7 cases, and fair in 4 cases, resulting in an excellent/good rate of 77.78%. Representative cases are illustrated in Fig. 2.

## Discussion

Avulsion fractures of the posterior calcaneal tuberosity were first documented by Mitchell et al.[6] in 1843, predominantly affecting elderly patients—particularly women in their seventh decade. Displaced fracture fragments frequently impinge upon the dorsal heel skin, precipitating localised soft-tissue necrosis and ulceration[22]. Beavis et al.[1] established a classification system based on anatomical characteristics, delineating three fracture patterns. Type II (beak-type) fractures represent the most common variant, featuring an oblique fracture line extending from the Achilles tendon insertion towards the calcaneal tuberosity shoulder, occasionally involving the posterior subtalar joint surface. This subtype carries the highest incidence of cutaneous complications among posterior tuberosity avulsions.

Management of posterior calcaneal tuberosity avulsion fractures demonstrates considerable therapeutic heterogeneity. Non-operative treatment is primarily indicated for minimally displaced fractures without significant soft-tissue compromise, and in elderly patients with functional impairment or reduced physiological reserve. Robb et al.[23] advocate conservative management for fractures displaced < 1 cm, involving immobilisation in a plantar flexion cast for 6–8 weeks. Notably, these treatment protocols require careful monitoring for possible adverse effects, including fracture displacement and pressure ulcers. Prolonged immobilization can also lead to risks such as joint stiffness and Achilles tendon contracture, potentially impacting functional recovery. Although fractures of the posterior calcaneal tuberosity are relatively rare, they often occur in patients with osteoporosis and other underlying medical conditions, which complicates clinical management. Surgical treatment of displaced fractures is challenging due to the strong pull of the Achilles tendon, especially in osteoporotic bone. While isolated cannulated screw fixation is commonly employed and offers considerable stability with minimal soft tissue dissection, it presents a not-insignificant risk of failure in osteoporotic bone due to screw cut-out and migration of the superior fragment [4, 5]. Gitajn et al. [4] reported a 38.5% failure rate with this technique, a finding supported by Khazen et al.'s biomechanical study[24] which demonstrated that solitary screw constructs withstand only approximately 250N of tensile force—significantly below physiological Achilles tendon loads.

This high failure rate has driven the development of many modified fixation techniques, each aiming to overcome the limitations of standard screw fixation. These include tension band wiring [25, 26]; screw-anchor combinations [27, 28]; fragment excision with double-row anchor fixation [29]; external fixation [30]; lag screws augmented by cast immobilization [5]; cannulated screws with cerclage wiring [31]; Krackow suture augmentation of screws [32]; plate-screw constructs [33]; and TightRope suspension techniques [34]. However, each method has specific limitations. For example, tension band wiring, while effective against traction forces, may cause prominent hardware that irritates soft tissue and footwear-related discomfort [25, 26]. Suture anchor-screw combinations improve biomechanical fixation strength [24], but might require extensive exposure for tendon weaving, increasing scarring risks [27, 28]. Other techniques, such as fragment excision or Ilizarov fixation [29, 30], pose concerns about poor bone-tendon healing, complex aftercare, or the requirement for plantar incisions that risk skin necrosis and painful scars [31, 34].

Consequently, rates of postoperative complications after surgical fixation of these fractures remain high, ranging from 30 to 70%, predominantly involving soft-tissue complications and implant failure [6, 35–38]. Mitchell et al. documented a reoperation rate of 35.9% [6], while Carnero-Martín et al. and Doany et al. reported rates of 38.1% and 35% (fixation failure), respectively [2, 22]. Takahashi et al. noted an overall 22% reoperation rate, with cannulated cancellous screw fixation alone exhibiting a 35% reoperation rate [7].

To address these challenges, we propose a novel construct combining Nice knots with cannulated screws for displaced Beavis Type II avulsion fractures in the elderly. Our technique involves the use of 1–2 cannulated screws placed perpendicular to the fracture line, supplemented by 1–2 Nice knot configurations, with placement tailored to the size of the fracture fragment. Key advantages include technical simplicity, high union rates, minimal soft-tissue complications, and reliable fixation strength permitting early rehabilitation. Retrospective analysis revealed a final mean AOFAS score of 85.94 ± 6.40, comparable to alternative techniques: Takahashi et al. reported 87 points for isolated cannulated screws [7]; Squires et al. documented 87.5 points in 22 tension band cases [26]. Regarding safety, our cohort exhibited a 16.67% complication rate, including delayed wound healing (n = 2, resolved with dressings) and periwound paraesthesia (n = 1, resolved after a 3-month mecobalamin regimen). Importantly, the cohort achieved a zero-reoperation rate, which is significantly lower than that reported in most comparable studies. This contrasts sharply with Gitajn et al.'s 35% and Yu et al.'s 10% reoperation rates, indicating superior clinical safety and efficacy [4, 35]. Despite these promising results, we recognise potential technical challenges. The learning curve for reliably tensioning the Nice knot should not be underestimated, as improper technique could theoretically result in knot slippage or loss of reduction. Moreover, although no cases of anchor pull-out or suture failure were observed during our short- to mid-term follow-up, the long-term integrity of the suture-anchor construct under continuous cyclic loading in osteoporotic bone remains a matter for further investigation. Critical technical considerations must be strictly adhered to: (1) Screw insertion site selection: Screws should be precisely implanted at the most prominent area of the posterior calcaneal tuberosity. This region offers higher cortical bone density and superior mechanical properties, significantly reducing fragmentation risk during insertion. The cortical bone at the Achilles tendon insertion site is dense and thick [39]. Athavale et al. confirmed optimal bone quality/strength in this zone [40]; Till et al. [41] similarly recommend screw fixation in the bone-rich posterior tuberosity for primary stability. (2) Screw parameters/orientation: Screw length must ensure full fracture penetration with perpendicular placement relative to the fracture line, engaging the plantar cortex without over-protrusion to establish robust biomechanical support. Wang et al.'s finite element analysis demonstrated that perpendicular fixation offers greater stability against axial and shear stresses [42]. (3) Nice knot application: Bone tunnels should be drilled adjacent to the plantar cortex to prevent cut-out in osteoporotic bone. The Nice knot—a bidirectional sliding configuration—delivers progressive tightening, anti-slip properties, dual-point fixation and high-tension maintenance [43]. Suture tails must be buried deep to the ventral Achilles insertion, avoiding skin contact. Collin et al. demonstrated significant resistance to knot elongation under dynamic loading, providing stable mechanical environments [44]. This technique meets fixation strength requirements while reducing stress-shielding when retained permanently, augmenting screw stability without traditional complications like Kirschner-wire migration or cable failure [45]. Although this technique offers certain benefits in terms of efficacy and safety, it also has several limitations. Precise control of screw placement and orientation depends largely on the surgeon’s anatomical knowledge and experience. Using intraoperative fluoroscopy during the initial adoption of the technique is advisable, with proficiency expected to improve as more cases are performed, which can reduce operative time and lower the risk of complications. The long-term stability should be carefully assessed in patients with severe osteoporosis or very small fracture fragments. During drilling and suture passing, special care must be taken to avoid injury to the medial and lateral cutaneous nerves of the heel. It is recommended to identify anatomical landmarks before beginning and maintain a safe distance during the procedure to minimise the risk of paraesthesia or neurological problems.

This study has several important limitations that must be acknowledged, many of which highlight avenues for future research, as rightly pointed out by the reviewers. First, the retrospective design, single-centre setting, and small sample size, while inherent to studying this uncommon fracture, limit the statistical power and generalisability of our findings. Second, we fully recognise the limitations of the primary outcome measure: the AOFAS score emphasises ankle motion (dorsiflexion/plantar flexion) and does not specifically evaluate subtalar joint function (inversion/eversion). Although assessment of tibiotalar motion remains clinically relevant due to stiffness from immobilisation, the lack of dedicated subtalar joint evaluation constitutes a methodological shortcoming. Furthermore, as a technical note, our study cannot objectively quantify the learning curve associated with the Nice knot technique. Future studies could record operative times and assess reduction quality across consecutive cases to define this curve. Lastly, the absence of biomechanical data comparing our construct to others prevents definitive claims regarding its mechanical superiority, and the follow-up period, while adequate for assessing union, may be insufficient to capture all long-term issues such as implant irritation, late-term failure, or subtalar arthritis. These limitations necessitate cautious interpretation of our results and highlight the need for prospective comparative studies with longer follow-up and more nuanced functional outcomes.

## Conclusion

This study provides preliminary feasibility data on using the Nice knot combined with cannulated screws for treating Beavis type II avulsion fractures of the posterior calcaneal tuberosity in osteoporotic elderly patients. The results demonstrate stable fixation, a low complication rate, and facilitated early functional rehabilitation. However, due to the retrospective design and the absence of a control group or statistical comparisons, these findings need further validation through prospective studies and biomechanical testing.

## Data Availability

The datasets used and/or analysed during the current study are available from the corresponding author upon reasonable request.
